# Comprehensive analysis of *WOX* genes uncovers that *WOX13* is involved in phytohormone-mediated fiber development in cotton

**DOI:** 10.1186/s12870-019-1892-x

**Published:** 2019-07-15

**Authors:** Peng He, Yuzhou Zhang, Hao Liu, Yi Yuan, Chan Wang, Jianing Yu, Guanghui Xiao

**Affiliations:** 1 College of Life Sciences, Shaanxi Normal University, Xi’an, 710119 China; 2 Zhengzhou Research Base, State Key Laboratory of Cotton Biology, Zhengzhou University, Zhengzhou, 450001 China; 3 The College of Life Sciences, Northwest University, Xi’an, 710069 China; 4 Key Laboratory of the Ministry of Education for Medicinal Plant Resources and Natural Pharmaceutical Chemistry, National Engineering Laboratory for Resouce Development of Endangered Crude Drugs in the Northwest of China, College of Life Sciences, Shaanxi Normal University, Xi’an, 710119 China

**Keywords:** *Gossypium hirsutum*, *WOX* genes, Expression analysis, Response regulator

## Abstract

**Background:**

The *WOX* (*WUSCHEL-RELATED HOMEOBOX*) gene family encodes a class of transcription factors that are unique to green plants, where they are involved in regulating the development of plant tissues and organs by determining cell fate. Although the importance of the *WOX* gene is well known, there are few studies describing their functions in cotton.

**Results:**

In this study, 32 WOX genes were found in *Gossypium hirsutum*. Phylogenetic analysis showed that WOX proteins of cotton can be divided into three clades: the ancient, intermediate, and WUS clades. The number of WOX proteins in the WUS clade was greater than the sum of the proteins in the other two clades. Our analysis revealed that 20 *GhWOX* genes are distributed on 16 cotton chromosomes and that duplication events are likely to have contributed to the expansion of the *GhWOX* family. All *GhWOX* genes have introns, and each GhWOX protein contains multiple motifs. RNA-seq data and real-time PCR showed that *GhWOX13* gene subfamily is specifically expressed at a high level in cotton fibers. We also identified putative GA, NAA, and BR response elements in the promoter regions of the *GhWOX13* genes and *GhWOX13* transcripts were significantly induced by GA, NAA, and BR.

**Conclusions:**

Our data provides a useful resource for future studies on the functional roles of cotton *WOX* genes and shows that the *GhWOX13* genes may influence cotton fiber development. Our results also provide an approach for identifying and characterizing WOX protein genes in other species.

**Electronic supplementary material:**

The online version of this article (10.1186/s12870-019-1892-x) contains supplementary material, which is available to authorized users.

## Background

The *WUSCHEL*-*RELATED HOMEOBOX* (*WOX*) gene family, which encodes one of the largest groups of transcription factors (TFs), is unique to plants, and belongs to a subclade of the homeodomain (HD) superfamily that can form a conserved DNA-binding homeo-domain [1]. Although *WOX* genes are spatiotemporally expressed during plant development, the number of *WOX* genes in the genomes of different plant species is not consistent. There are 15, 11, and 21 *WOX* gene family members in *Arabidopsis*, sorghum, and maize, respectively [2–4]. Phylogenetic analyses of WOX proteins from multiple higher plant species such as *Arabidopsis*, sorghum, maize, and rice show that they cluster into three clades, known as the WUS clade, the intermediate clade, and the ancient clade. However, the lower plants (green algae and moss) only contain the ancient type of the WOX proteins [5]. In *Arabidopsis*, 15 WOX proteins can be divided into three phylogenetic clades, AtWOX1–7 and AtWUS belonging to the WUS clade, AtWOX8, AtWOX9, AtWOX11, and AtWOX12 belonging to the intermediate clade, AtWOX10, AtWOX13, and AtWOX14 belonging to the ancient clade [6, 7].

The WOX proteins have been shown to act a pivotal part in numerous developmental processes, such as organ formation, embryo patterning, stem cell maintenance [8–10]. *AtWOX1* is highly expressed in the initiating vascular primordium of the cotyledons and emerging leaves, and over-expression of *AtWOX1* caused small leaves, dwarf plant and low fertility [11, 12]. Ectopically over-expression of *TaSF (STENTOFOLIA)*, the ortholog of *AtWOX1*, results in transgenic plants with broadened leaves, accelerated flowering and increased chlorophyll content [13]. *WOX3* has a highly conserved function during the recruitment of founder cells to form the lateral domains of vegetative and floral organs in *Arabidopsis* and maize. A loss-of-function *AtWOX3* mutant exhibited a degeneration of the lateral stipules and sepals [14]. *AtWOX4* is required for auxin-dependent procambium differentiation and/or maintenance, which shows that the *WOX* gene family influences the auxin-dependent regulation of lateral plant growth [15, 16]. Rice *WOX4* is responsible for meristem maintenance and reduction of *OsWOX4* expression led to severe malformed leaf primordia [17]. *AtWOX5*, which is induced by auxin, is a negative factor to regulate IAA homeostasis to maintain stem cells in root apical meristem (RAM) [18]. In *Populus*, *WOX5a* was mainly expressed in the adventitious root (AR) tip and lateral root tip. Over-expression of *PtoWOX5a* led to increased AR number, decreased AR length and leaf number [19]. Also, over-expression of *WOX11* and *WOX12* significantly increased AR number and decreased ectopic roots in poplar [20]. A homolog of AtWOX11, *Os*WOX11, interacts with H3K27me3 demethylase JMJ705 to promote the expression of downstream gene during shoot development [21]. In addition, *OsWOX11* is also involved in lateral root initiation, root hair formation, and responses to abiotic stresses [22, 23].

Cotton is the important economic crop in the world, which can be used for textile. Cotton fiber is the single seed coat epidermal cell. Quality and yield of fiber depends mainly on three biological processes; fiber initiation, fiber elongation, and secondary cell wall deposition [24–26]. Many studies have focused on fiber cell growth in cotton. So far, genome sequences of three cotton species have been completed, including the allotetraploid *G. hirsutum*. (AADD, 2n = 52) and the two diploid progenitors *G. arboreum* (AA; 2n = 26) and *G. raimondii* (DD, 2n = 26) [27–31]. These genome sequences have enabled the identification of *WOX* genes at a genome-wide scale in cotton.

The roles of WOX proteins have been well-documented in *Arabidopsis*, rice, and maize, however, the functions of WOX proteins in cotton, especially in fiber development, are largely unknown. In this study, we identified 32 cotton *WOX* genes and found that they cluster into the previously-known ancient, WUS, and intermediate phylogenetic clades. Chromosomal distribution analysis revealed that 20 *GhWOX* genes are distributed on 16 cotton chromosomes. Analysis of gene expression patterns showed that *GhWOX13* genes are specifically expressed at high levels in the cotton fiber. In addition, we found that all of the *GhWOX13* genes had putative GA, NAA, and BR response elements in their promoter regions and can be induced by these hormones. Taken together, the results of our study provide a useful resource for future studies on the functional roles of cotton *WOX* genes, and demonstrated that the *GhWOX13* gene products may act to influence cotton fiber development. We also provide an approach for identifying and characterizing WOX proteins in other plant species.

## Results

### Identification of *WOX* gene family proteins in *Gossypium*

To identify *WOX* family members in *Gossypium*, 15 AtWOX protein sequences were used as queries for blast searching of the cotton genome database with the E-value cutoff of 0.001. The candidate GhWOX proteins were manually inspected to ascertain that the candidates contained the conserved DNA-binding homeodomain using the InterPro protein sequence analysis & classification tool. As a result, we identified 32, 18, and 19 *WOX* protein coding genes in *G. hirsutum*, *G. arboreum*, and *G. raimondii*, respectively (Table 1). We found that the number of *WOX* genes identified in the tetraploid *G. hirsutum* was lower than the combined number from its diploid progenitors suggesting some loss after polyploid formation. Compared with *Arabidopsis*, multiple *WOX* genes, such as *WOX3, WOX4*, *WOX13* and *WUS*, were expanded in *Gossypium*. On the contrary, *WOX7* and *WOX8* were missing in three cottons, whereas they were found in *Arabidopsis*, suggesting that *WOX7* and *WOX8* genes may have been lost in *Gossypium* after they diverged from the common ancestor of *Gossypium* and *Arabidopsis*.

**Table 1:** The number of *WOX* gene family members in the *G. hirsutum*, *G. raimondii*, *G. arboreum*, and *A. thaliana* genomes

Species	*G. hirsutum*	*G. raimondii*	*G. arboreum*	*A. thaliana*
WOX1	1	1	1	1
WOX2	4	1	2	1
WOX3	4	2	2	1
WOX4	6	3	2	1
WOX5	2	1	1	1
WOX6	1	1	0	1
WOX7	0	0	0	1
WOX8	0	0	0	1
WOX9	1	2	2	1
WOX10	1	0	1	1
WOX11	1	1	1	1
WOX12	2	1	1	1
WOX13	4	2	2	1
WOX14	1	2	1	1
WUS	4	2	2	1
Total	32	19	18	15

### Phylogenetic relationships of the WOX proteins from *G. hirsutum*, *G. arboreum*, *G. raimondii*, and *A. thaliana*

**Fig. 1:**
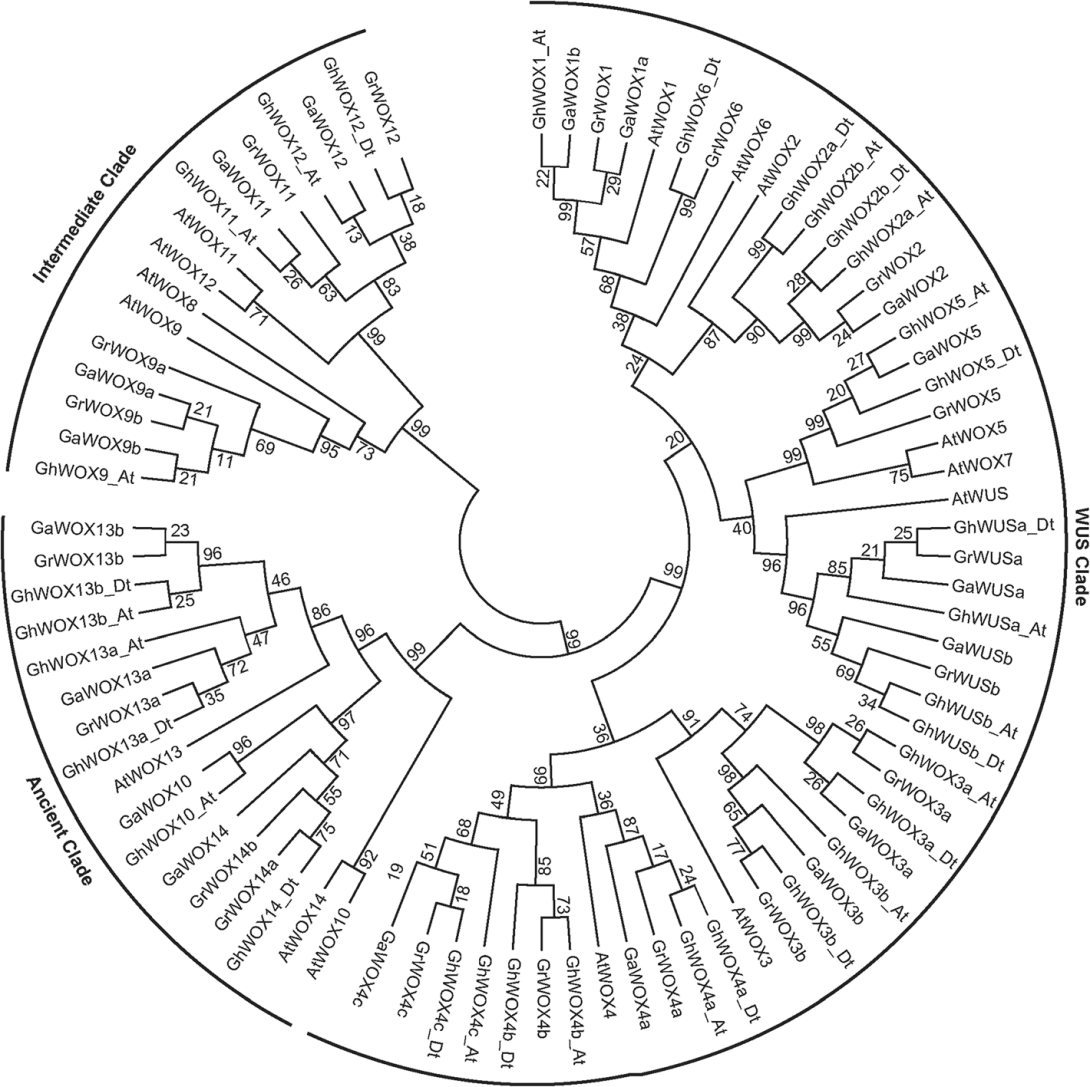
An unrooted Neighbor-Joining tree showing phylogenetic relationships among the predicted WOX proteins from *Gossypium hirsutum* (*Gh*), *Gossypium arboreum* (*Ga*), *Gossypium raimondii* (*Gr*) and *Arabidopsis thaliana* (*At*). The phylogenetic tree was constructed using MEGA 6.0, and the WOX proteins from the four plant species group into three clades (ancient, WUS, and intermediate)

To evaluate the evolutionary and phylogenetic relationships of the *WOX* gene families among species, a total of 89 WOX protein sequences, including 32 GhWOXs, 18 GaWOXs, 19 GrWOXs, and 15 AtWOXs sequences, were used to construct an unrooted phylogenetic tree based on the alignment using the Neighbor-Joining (NJ) method as implemented in MEGA 6.0 with 1000 bootstrap replicates (Fig. 1). According to the phylogenetic relationship of WOX family, the GhWOX family proteins were also divided into three clades: the WUS clade, the ancient clade and the intermediate clade, The WUS clade, which was the largest clade, contains 23 GhWOX proteins. The ancient clade has six GhWOX proteins. The remaining members belong to the intermediate clade.

### Chromosomal locations of the *GhWOX* genes

**Fig. 2:**
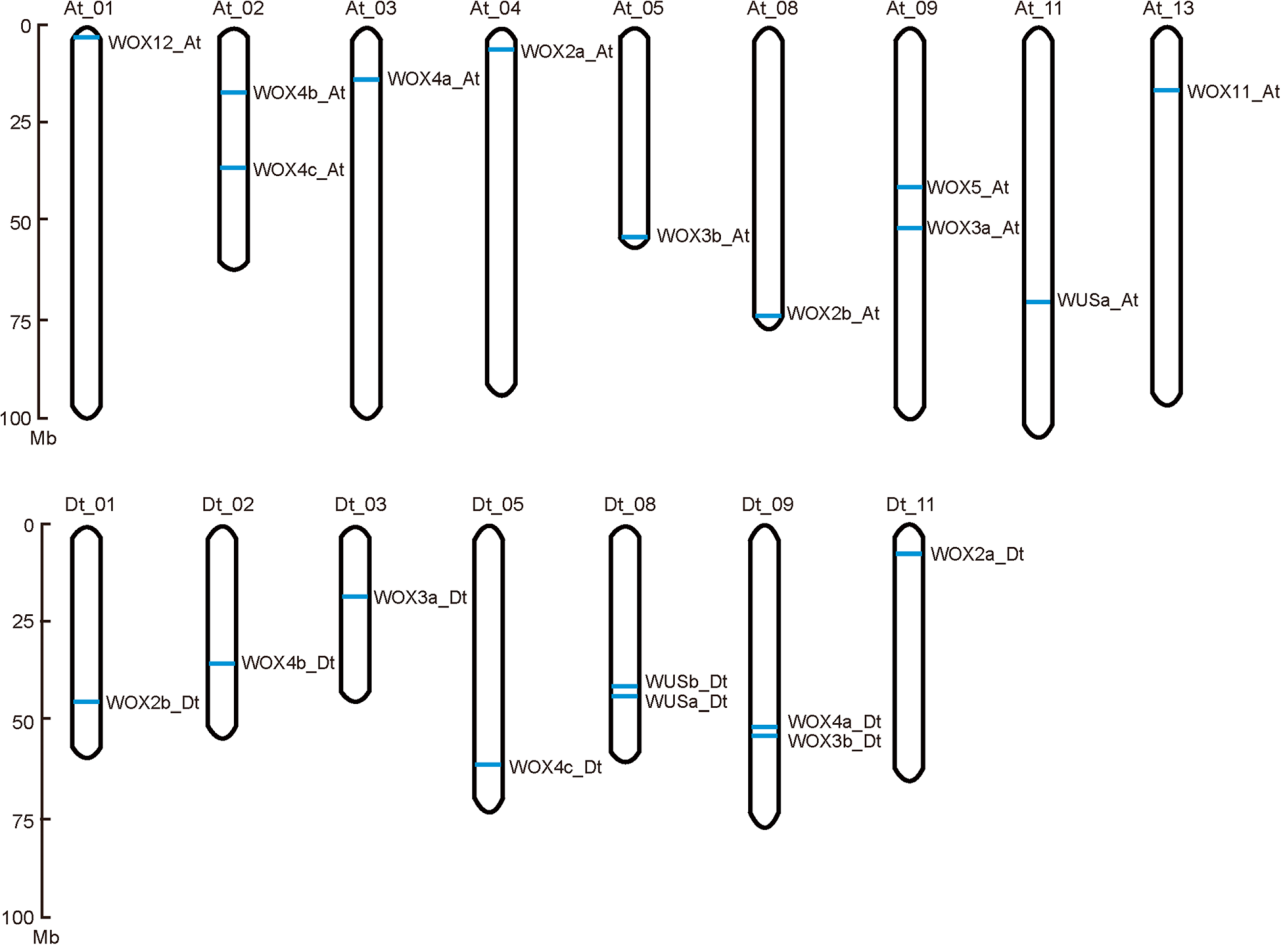
Chromosomal distributions of the identified *GhWOX* genes. Chromosomal locations are shown from top to bottom on corresponding chromosomes based on the *Gossypium hirsutum* acc. ‘Texas Marker-1’ (TM-1) genome annotation v1.0

We investigated the chromosomal location of the 32 *GhWOX* genes based on the annotation of the *G. hirsutum* genome. Genomic localization of the *GhWOX* genes showed that 20 *GhWOX* genes map to 16 chromosomes, while 12 *GhWOX* genes map to scaffold sequences that are not assigned to chromosomes (Fig. 2). Cotton chromosomes At_02, At_09, Dt_08, and Dt_09 harbor two *GhWOX* genes each, while all other chromosomes have only one. However, no *GhWOX* genes were found on chromosomes At_06, At_07, At_10, At_12, Dt_04, Dt_06, Dt_07, Dt_12, and Dt_13. We analyzed duplication events in the *GhWOX* genes mapped to chromosomes, and identified three types of gene duplication: tandem duplication, dispersed duplication, and segmental duplication (Additional file 2: Table S2). Except for *GhWOX12_At* and *GhWUS1b_Dt* which were derived from dispersed and tandem duplications, respectively, the other mapped *GhWOX* genes all resulted from segmental duplications This result indicated that segmental duplications were the main contributors to the expansion of *GhWOX* genes in *G. hirsutum*.

### *GhWOX* gene structure and domain analysis

**Fig. 3:**
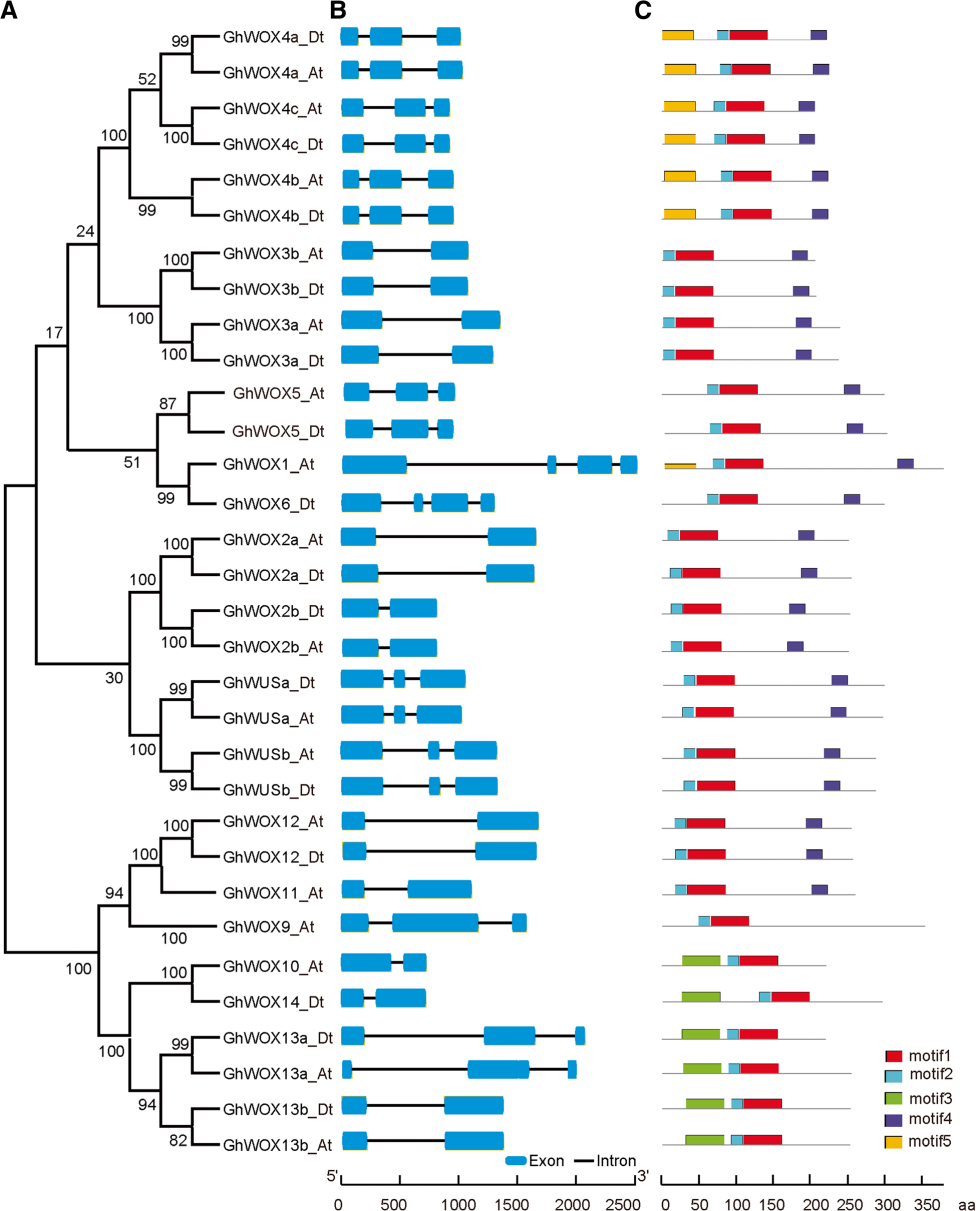
Phylogenetic relationships, gene structure, and domain compositions of the *GhWOX* proteins. **a** A phylogenetic tree was constructed with MEGA 6.0 using the Neighbor-Joining (NJ) method with 1000 bootstrap replicates based on a multiple alignment of the amino acid sequences of the GhWOX proteins. **b** Exon/intron structures of the *GhWOX* genes. Exons and introns are represented by blue boxes and black lines, respectively. **c** Schematic diagram showing the conserved domains in the GhWOX proteins as annotated by InterPro (http://www.ebi.ac.uk/ interpro/scan.html). Different color boxes represent different conserved protein motifs

In order to better understand and explore the diversity of the *WOX* genes in *G. hirsutum*, we analyzed the conserved motifs and exon/intron structures of the *GhWOX* genes. The gene structures of *GhWOX* was generated by aligning the cDNA sequences and corresponding genomic DNA sequences. An unrooted phylogenetic tree was obtained with predicted GhWOX protein sequences (Fig. 3a). Exon/intron structure analysis showed that most *GhWOX* genes contain two introns, and the others contain no less than one intron, suggesting that *GhWOX* gene structure is conserved (Fig. 3b). We also analyzed the conserved motifs of the GhWOX proteins using the InterPro protein sequence analysis server, and five distinct conserved motifs were identified. The composition patterns of the motifs tend to be consistent with the phylogenetic tree. Motifs 1 and 2 are present in all GhWOX protein subfamilies, motif 4 is present in 24 GhWOX proteins except for GhWOX9, GhWOX10, GhWOX13, and GhWOX14. Motif 3 is present in GhWOX10, GhWOX13, and GhWOX14, and motif 5 is found only in GhWOX1 and all members of the GhWOX4 subfamily (Fig. 3c). The GhWOX proteins from the ancient clade contain three identical motifs, which could indicate that the proteins in this clade have the same function, and that the functions are redundant. In addition, a new motif was found that is specific to the GhWOX1_At protein, although it shared three identical motifs with GhWOX6A. The two proteins are very close in the phylogenetic tree, suggesting that they may have a shared function. However, a new motif could contribute a novel function to the GhWOX1_At protein.

### Expression patterns of the *GhWOX* genes in various tissues

**Fig. 4:**
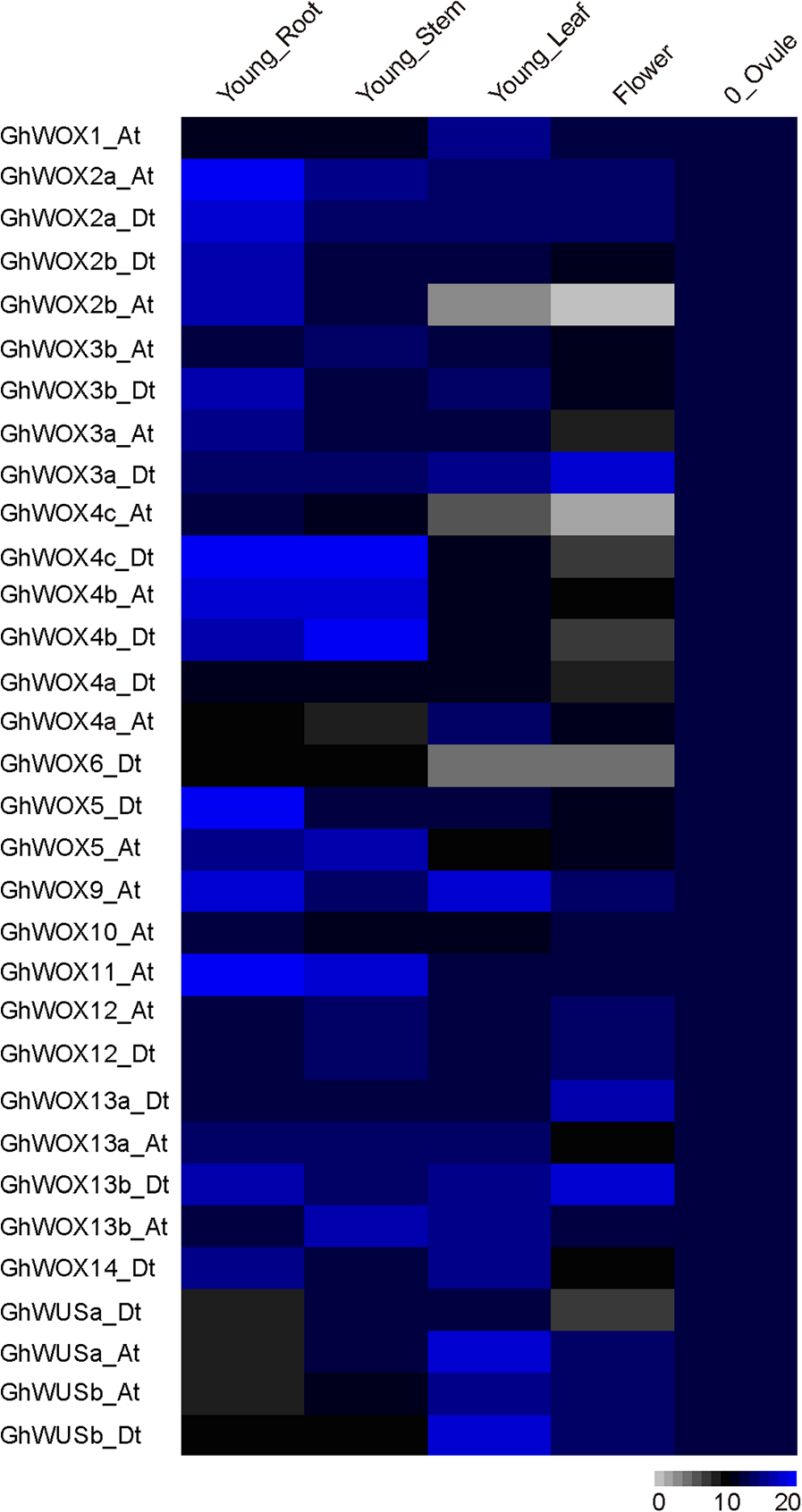
Heat map showing the expression patterns of the *GhWOX* genes in five different tissues of cotton (*Gossypium hirsutum* ‘Xuzhou 142’)

To investigate the tissue-specific expression profiles of 32 *GhWOX* genes, their relative expression levels in different organs, including young roots, young stems, young leaves, flowers, and 0 dpa ovules were analyzed from RNA-seq data. As shown in Fig. 4, the data indicate that expression of the *GhWOX* genes varies in the different organs and tissues; *GhWOX2*, *GhWOX4*, *GhWOX5*, *GhWOX9*, *GhWOX11*, *and GhWOX13b_Dt* are highly expressed in young roots, *GhWOX4*, *GhWOX5*, *GhWOX11*, *and GhWOX13b_At* are highly expressed in young stems, and *GhWOX9_At, GhWUSa_At*, *and GhWUSb_Dt* are highly expressed in young leaves, implying that the *GhWOX* genes have different roles in the growth and development of cotton. These results are consistent with those of previous reports on the function of individual *WOX* genes, which suggests that *WOX* gene functions are conserved in different species. Interestingly, all of the *GhWOX* genes have nearly identical expression levels in the ovules.

**Fig. 5:**
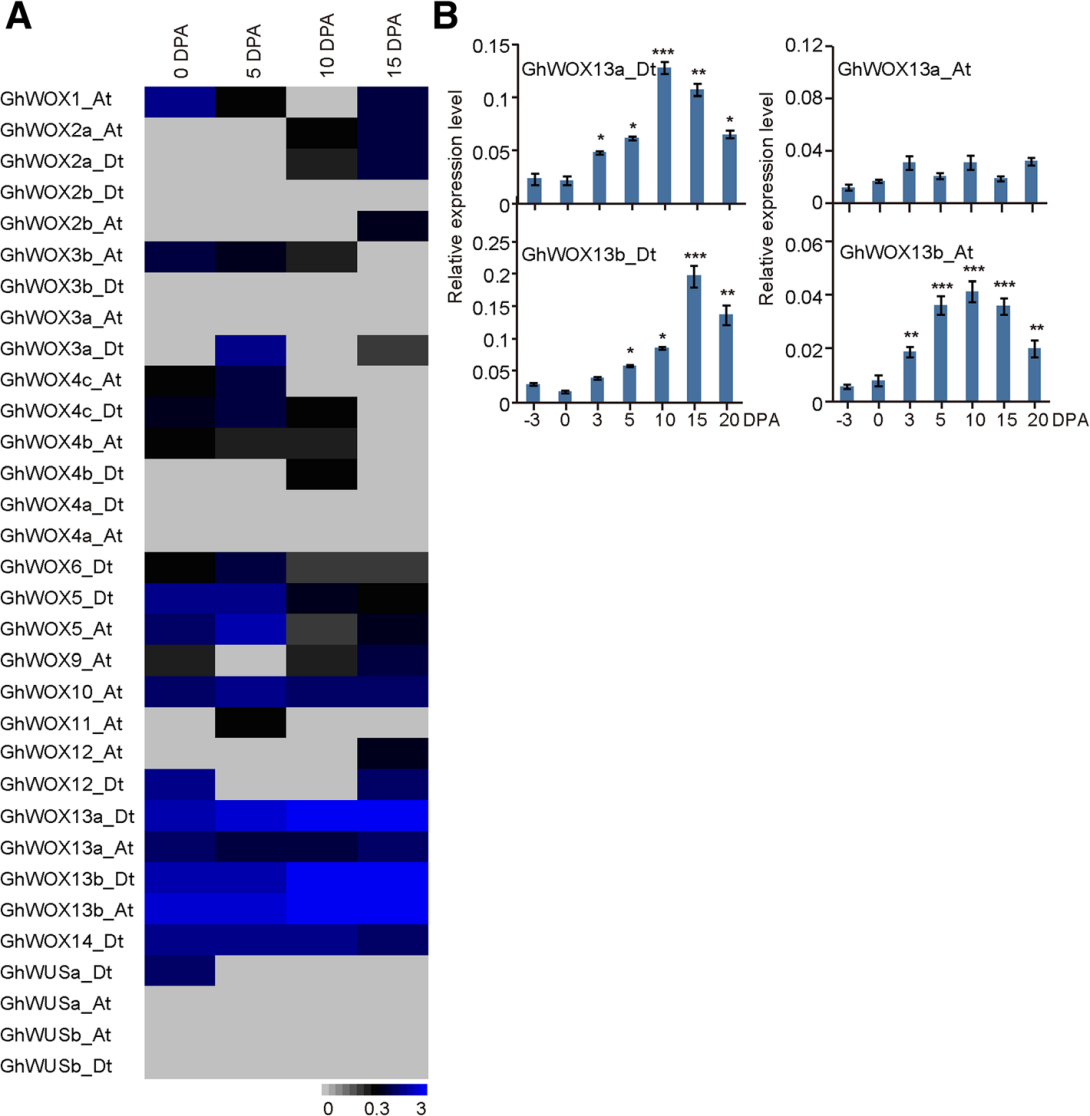
RNA-seq and qRT-PCR analysis of *GhWOX* genes during different stages of fiber development. **a** Heat map showing the relative expression levels of the *GhWOX* genes during different stages of fiber growth based on RNA-seq data. **b** Expression profiling of the *GhWOX13* subfamily genes during cotton fiber development. Gene expression data were obtained from qRT-PCR assays with three independent replicates. The *G. hirsutum* ubiquitin gene *GhUBQ7* was used to normalize the expression of the *GhWOX* genes

We further investigated the expression profiles of *GhWOX* genes and found that none of them displayed a significant change during the fiber development stage except for *GhWOX13a_Dt*, *GhWOX13b_At*, and *GhWOX13b_D*., which were highly expressed in fibers at 10 and 15 dpa (Fig. 5a). Furthermore, we verified the RNA-seq data using quantitative real-time PCR (qRT-PCR) experiment. As a result, *GhWOX13* were consistent with the RNA-seq data. The transcripts of *GhWOX13a_Dt* and *GhWOX13b_At* gradually increased and reached a peak in the fibers at 10 dpa (Fig. 5b). These results indicate that *GhWOX13* genes may play an important role in cotton fiber development.

### *GhWOX13* gene expression is stimulated by GA, BR, and auxin

**Fig. 6:**
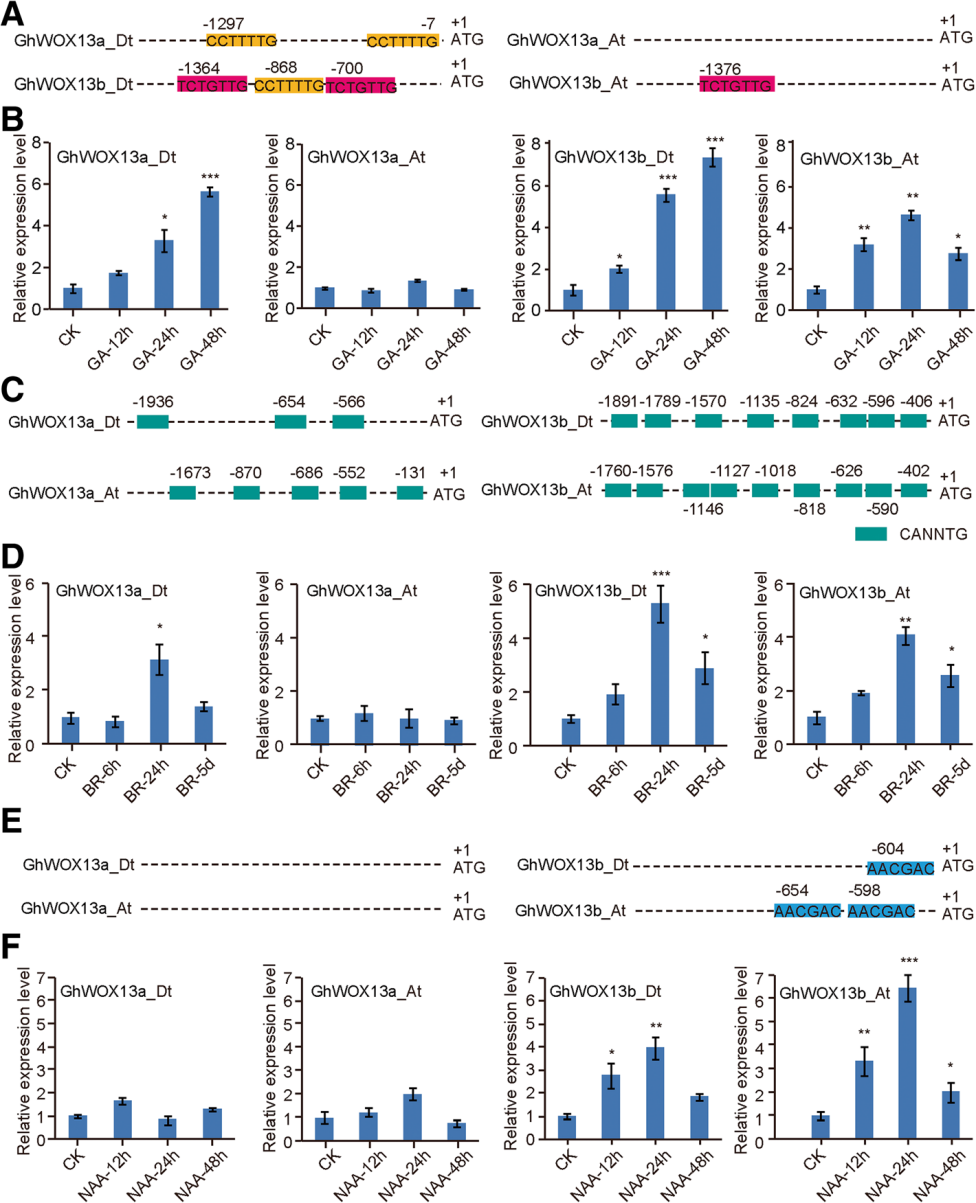
*GhWOX13* subfamily gene transcription is induced by gibberellic acid (GA), brassinosteroid (BR), and auxin treatments. **a** Identification of putative auxin response elements (AuxREs) in the promoter regions of the four *GhWOX13* gene family members. **b** GA-induced transcription in the three *GhWOX13* genes with AuxREs in their promoter regions. The promoter of *GhWOX13B* does not contain an AuxRE. (**c**) Identification of E-box elements in the promoter regions of *GhWOX13a_At/13a_Dt/13b_At/13b_Dt*. **d** Transcription of the four *hWOX13* family genes after BR treatment for 6 h, 24 h, and 5 d. **e** Identification of putative auxin response TGA elements in the promoter regions of the *GhWOX13b_At/13b_Dt* genes. **f** Transcription of the four *GhWOX13* family genes after treatment with NAA for 12, 24, and 48 h. *GhWOX13b_At* and *GhWOX13b_Dt* have putative TGA elements in their promoter regions. Gene expression data were obtained from qRT-PCR assays with three replicates per sample, with each replicate performed using independent materials. The relative expression levels of each gene were determined by normalizing to the expression level in the CK ovules, which was set to 1.0. Statistical significance was determined using one-way analysis of variance combined with Tukey’s test. *, *P* < 0.05; **, *P* < 0.01; ***, *P* < 0.001

In order to identify the putative regulatory elements that control expression of the *WOX* genes in cotton, we analyzed the promoter regions of the *GhWOX* genes. We found that most of the *GhWOX* gene promoters contained at least one putative GA-response element (i.e. GARE motif, P-box, and TATC-box) except for *GhWOX1_At*, *GhWOX3a_At*, *GhWOX3b_At*, *GhWOX3b_Dt*, *GhWOX4b_Dt*, *GhWOX4c_At, GhWOX12_Dt*, *GhWOX13a_Dt*, and *GhWOX14_Dt* (Additional file 1: Figure S1). We next analyzed the putative BR-response element and found that all *GhWOX* genes had putative BR-response elements in their promoter regions (Additional file 1: Figure S2). We also searched for putative auxin-related *cis*-elements in the promoter regions of *GhWOX* genes, and found that *GhWOX3a_Dt*, *GhWOX4b_Dt*, *GhWOX4c_At*, *GhWOX5_Dt*, *GhWOX9_At*, *GhWOX13b_At* and *GhWOX3b_Dt* contain a putative auxin response element (AuxRE) in their upstream promoter regions (Additional file 1: Figure S3). Since the *GhWOX13* genes are specifically expressed in cotton fibers, we analyzed the promoter regions of the *GhWOX13* genes and identified putative GA, NAA, and BR responsive *cis*-elements in the promoters (Fig. 6a, c and e). In order to determine whether *GhWOX13* genes are induced by GA, NAA, and BR, the mRNA levels of *GhWOX13a_At*, *GhWOX13a_Dt*, *GhWOX13b_At*, and *GhWOX13b_Dt* were evaluated by qRT-PCR after treatment with the three phytohormones. The results showed that the expression of *GhWOX13a_Dt* and *GhWOX13b_Dt* was up-regulated after treatment with GA for 48 h, the *GhWOX13b_At* mRNA level was significantly increased after treatment with GA for 24 h, but the transcription of *GhWOX13a_At* showed no change in response to GA treatment (Fig. 6b). *GhWOX13a_Dt*, *GhWOX13b_At* and *GhWOX13b_Dt* were up-regulated, with expression peaking after 24 h of BR treatment, while transcription of *GhWOX13a_At* showed no response to BR (Fig. 6d). Expression of *GhWOX13b_At* and *GhWOX13b_Dt* was induced by NAA, while the expression of *GhWOX13a_At*, *GhWOX13a_Dt* showed no effect (Fig. 6f). These results indicate that the *GhWOX13* genes may play an important role in cotton fiber elongation mediated by plant hormones.

## Discussion

Cotton is the one of the most significant economic crops cultivated worldwide, and it is an important source of fiber and oil. Whole genome sequencing of the *G. arboreum*, *G. raimondii*, and *G. hirsutum* genomes has been completed recently, and a number of genes associated with fiber development and leaf formation have been discovered by combining genome analysis with high-throughput transcriptome sequencing. However, the functions of the *WOX* genes in cotton, especially as they relate to fiber development, are largely unknown. In this study, we identified 32, 18, and 19 *WOX* genes in *G. hirsutum*, *G. arboreum*, and *G. raimondii*, respectively. Although the total number of *WOX* genes in *G. hirsutum* is much higher than in the other two species, the number of genes in the At and Dt genomes of *G. hirsutum* are less than the numbers in *G. arboreum* or *G. raimondii*. Gene copy numbers were largely reduced during the cotton-specific polyploidization event [32–34]. Our results are consistent with the finding that the rate of gene loss in allotetraploid cotton was higher than that in the two ancestral diploid species. Previous research has shown that *WOX* gene family can be divided into three major clades; the ancient clade which is present in green algae and all lineages of land plants, while intermediate and WUS clades are only found in ferns and seed plants [35]. In this study we identified 32 *WOX* genes representing all three major clades in the allotetraploid cotton species *G. hirsutum*. Yang et al. identified *WOX* gene family members in *Upland cotton and* also found that *WOX* genes could be naturally classified into three clades [36], which is consistent with our finding. Four of the *GhWOX*s belonged to the intermediate clade and six belonged to the ancient clade. The number of genes in the WUS clade was much higher than in either the intermediate or ancient clades, which is consistent with a previous report [37]. In *A. thaliana*, there are 15 AtWOX proteins, including four in the intermediate clade, three in the ancient clade, and eight in the WUS clade.

Gene structure analysis predicted that 15 *GhWOX* genes contain one intron, 15 *GhWOX* genes contain two introns, and two *GhWOX* genes (*GhWOX1_At* and *GhWOX6_Dt*) contain three introns. *GhWOX1_At* and *GhWOX6_Dt* have nearly identical gene structures within the *WOX1/6* subgroup, although the intron phases and lengths differ. Similarly, the *Arabidopsis* genes *AtWOX1* and *AtWO*X6 are in one phylogenetic clade and share a similar gene structure with three introns. Protein sequence motif analysis revealed that all GhWOX subfamilies contain motifs 1 and 2. Motif 3 is only found in GhWOX10, GhWOX13, and GhWOX14, and these proteins belong to the ancient clade (Fig. 1), indicating that motif 3 is conserved in the most ancient group of WOX proteins. These results suggest that the specific functions of proteins in the different subfamilies may be due to the presence of specific sequence motifs. The patterns of introns and motifs, which correlate well with the phylogenetic clades, strongly support the close evolutionary relationships among the *GhWOX* genes within each of the subfamilies.

The WOX proteins have been shown to act a pivotal part in numerous developmental processes, such as organ formation, embryo patterning, stem cell maintenance [38, 39]. Our data show that many *GhWOX* genes have different expression patterns with the homologous genes in *Arabidopsis*. For example, in contrast to *WOX2* expression in the *Arabidopsis* embryo, we found that *GhWOX2a_At*/*2a*_Dt/*2b*_At/*2b*_Dt*,* are specifically expressed in the young root, and exhibit an expression profile similar to that of *WOX2* in *Populus tomentosa*, which is mainly expressed in the roots [40]. We also noted that the duplicated genes show varying expression levels; *GhWOX13a_Dt* and *GhWOX13b_Dt* are primarily expressed in flowers, while *GhWOX13b_At* is expressed at a high level in young stems. These results suggest that some of *WOX* genes might acquire diverse functions during evolution.

*GhWOX13a_Dt*, *GhWOX13b_At*, and *GhWOX13b_Dt* are highly expressed in the cotton fiber, and their expressions gradually increase during fiber elongation. We analyzed the promoters of the *GhWOX13* genes and identified putative GA, NAA, and BR response *cis*-elements in the promoters; we also found that the *GhWOX13* genes are up-regulated after treatment with GA, BR, or NAA. *AtWOX13* have high expression levels in inflorescences, floral buds, emerging lateral roots and root tip [41, 42]. The *wox13* mutant showed slightly wider fruits with a reduced number of lateral roots in *Arabidopsis*. Our data indicate that *GhWOX13* genes may involved in phytohormone mediating fiber elongation in cotton.

Taken together, our work not only comprehensively identified the *WOXs* in *G. hirsutum*, but also found that *GhWOX13* transcripts were expressed during cotton fiber development and can be significantly induced by GA, NAA, and BR. Our research provides a clue for further uncovering the precise roles of *WOX* genes in cotton fiber development.

## Conclusions

*Gossypium hirsutum* contained 32 WOX proteins and can be divided into three clades: the ancient, intermediate, and WUS clades. The number of WOX proteins in the WUS clade was more than that in the remaining two clades. Multiple introns and motifs were found in *GhWOX* genes. *GhWOX13* genes were specifically and highly expressed in cotton fiber cells and they transcripts can be induced by GA, NAA, and BR. Our results also provide an approach for identifying and characterizing *WOX* genes in other species.

## Methods

### Databases and sequences

The genome sequence of *Arabidopsis thaliana* was downloaded from the TAIR database (http://www.arabidopsis.org/). The genome sequences of *G. arboreum* (A2, CRI-Updated assembly v1.0), *Gossypium raimondii* (D5, JGI assembly v2.0), and *G. hirsutum* (AD1, NAU assembly v1.1) were downloaded from the CottonFGD database (https://cottonfgd.org/). We used Ga (*G. arboreum*), Gr (*Gossypium raimondii*), Gh (*G. hirsutum*), and At (*A. thaliana*) as prefixes in the naming of *WOX* genes in this study.

### Promoter analysis, multiple sequence alignment, and phylogenetic analysis

Sequences of 2000 nucleotides prior to the start codon were extracted from the genomic sequences of the *GhWOX* genes, and the *cis*-acting regulatory elements were predicted by the program PlantCARE online (http://bioinformatics.psb.ugent.be/webtools/plantcare/html/). The gene IDs of the *WOX* genes from the above three species are shown in Additional file 2: Table S1. The full predicted amino acid sequences of the *WOX* genes from the four plant species were aligned using ClustalW. A Neighbor-Joining (NJ) phylogenetic tree was constructed by MEGA 6.0 software with pairwise deletion option, poisson correction model and uniform rates. Bootstrap test were carried out with 1000 replicates for evaluating the statistical reliability of the phylogenetic tree [43].

### Gene structure, protein motif analysis, and genomic location

The Gene Structure Display Server (GSDS, http://gsds.cbi.pku.edu.cn/) was used to draw the exon–intron structures of *GhWOX* genes based on the full-length genomic sequences and the corresponding coding sequences obtained as described above [44]. InterPro, protein sequence analysis & classification (http://www.ebi.ac.uk/interpro/scan.html), was used to identify conserved domains in the GhWOX proteins [45]. Chromosomal position information of each *GhWOX* gene was acquired from the genome annotation, and the relative locations were shown on the respective chromosomes in a top-to-bottom orientation.

### Plant materials and hormonal treatments

*Gossypium hirsutum* (‘Xuzhou 142’) seed was obtained from Institute of Cotton Research of Chinese Academy of Agricultural Sciences (Anyang, China). Cotton plants were grown in a climate-controlled greenhouse with a 16 h light/8 h dark photoperiod at 30 °C. Fresh cotton seed fibers were harvested from bolls at various days post anthesis (DPA) at the indicated time points and then immediately frozen in liquid nitrogen as previously reported [46].

The plant hormone treatments were performed using previously-described methods [46]. Briefly, self-pollinated cotton flowers were collected at 1 DPA (days post anthesis). Subsequently, bracts, sepals and petals were removed and the ovaries were sterilized using 10% hypochlorous acid for 15 min, and then rinsed with sterile distilled water for seven times. The ovules were carefully dissected from the ovaries under sterile conditions and immediately floated on the liquid medium (0.272 g/L KH_2_PO_4_, 6.183 mg/L H_3_BO_3_, 0.242 mg/L Na_2_MoO_4_·2H_2_O, 0.441 g/L CaCl_2_·2H_2_O, 0.83 mg/L KI, 0.024 mg/L CoCl_2_·6H_2_O, 0.493 g/L MgSO_4_·7H_2_O, 16.902 mg/L MnSO_4_·H_2_O, 8.627 mg/L ZnSO_4_·7H_2_O, 0.025 mg/L CuSO_4_·5H_2_O, 5.055 mg/L KNO_3_, 8.341 mg/L FeSO_4_·7H_2_O, 11.167 mg/L Na_2_EDTA, 0.492 mg/L Nicotinic acid, 0.822 mg/L Pyridoxine·HCl, 1.349 mg/L Thiamine·HCl, 0.180 g/L Myo-inositol, 18.016 g/L D-Glucose, 3.603 g/L D-Fructose) containing 1 μM GA (Gibberellic acid), 5 μM BR (Brassinosteroid), or 5 μM NAA (1-Naphthaleneacetic acid) in a conical flask, respectively [46]. Then the ovules were cultured at 30 °C in darkness without agitation. For GA and NAA treatment, ovules incubated for 12 h, 24 h, and 48 h were collected together with fibers because of the difficulty of separating them from each other. For BR treatment, 6 h, 24 h, and 5 d ovules were sampled together with fibers. The samples were frozen in liquid nitrogen immediately after collection and stored at − 80 °C prior to use in the experiments. For each treatment, three individual samples were collected and the analysis was performed on the three biological replicates. GA, BR, and NAA were purchased from Sigma-Aldrich.

### RNA extraction and qRT-PCR analysis

Total RNA was extracted using the E.Z.N.A.® Plant RNA Kit (OMEGA) according to the manufacturer’s instructions. The tissues were disrupted and homogenized. The column flow-through at the very last step was mixed with the membrane-binding solution and then loaded into the HiBind RNA Mini column. Finally, RNA was washed with RWC buffer and RNA wash buffer to remove proteins, polysaccharides, and salts. The total RNA was treated with DNaseI to remove gDNA contamination. First-strand cDNA was then synthesized using the PrimeScript RT Reagent Kit following the manufacturer’s protocol (TaKaRa, Dalian, China).

The qRT-PCR assays were performed using a Bio-Rad Real Time PCR detection system (Bio-Rad CFX96 Touch) [47]. The SYBR Green qRT-PCR reactions contained 10 μl SYBR® Premix Ex Taq™ II (Takara), 0.5 μl of 10 μM primers, and 2 ng cDNA template. The final volume was adjusted to 20 μl with ddH_2_O. The PCR cycling conditions were 95 °C for 30 s, followed by 40 cycles of 95 °C for 5 s and 60 °C for 30 s. Relative gene expression was calculated using the 2^−ΔΔCt^ method [47]. The experiment was performed with three biological replicates and each biological replicate was performed with three technical replicates. Primers used for qRT-PCR analysis are shown in Additional file 2: Table S3. The cotton ubiquitin gene *GhUBQ7* was used as the internal control for normalization of gene expression in each qRT-PCR experiment.

## Additional files

Additional file 1: **Figure S1.** Identification of putative GA-related *cis*-elements in the promoter regions of the *GhWOX* genes. **Figure S2.** Putative BR response elements identified in the *GhWOX* gene promoter regions. **Figure S3.** Detection of putative auxin-related *cis*-elements in the *GhWOX* gene promoter regions. (DOCX 3317 kb)

Additional file 2: **Table S1.** Analysis of the *G. hirsutum WOX* gene family members and their orthologs in the AA and DD genome cotton species. **Table S2.** Analysis of duplication events in the *G. hirsutum WOX* genes mapped to chromosomes. **Table S3.** PCR primers used for analysis of duplication events in the *G. hirsutum WOX13* gene family members mapped to chromosomes. (DOC 107 kb)

## Data Availability

All of the data and materials supporting our research findings are contained in the methods section of the manuscript. Details are provided in the attached supplementary data.

## Abbreviations

BR: Brassinosteroids
DPA: Days post anthesis
GA: gibberellin
NAA: Naphthaleneacetic Acid
WOX: WUSCHEL-RELATED HOMEOBOX
