# Supplementary material for: Comprehensive analysis of WOX genes uncovers that WOX13 is involved in phytohormone-mediated fiber development in cotton
Source: BMC Plant Biol. 2019 Jul 15;19:312. doi: 10.1186/s12870-019-1892-x (PMC6632001; doi:10.1186/s12870-019-1892-x)

**
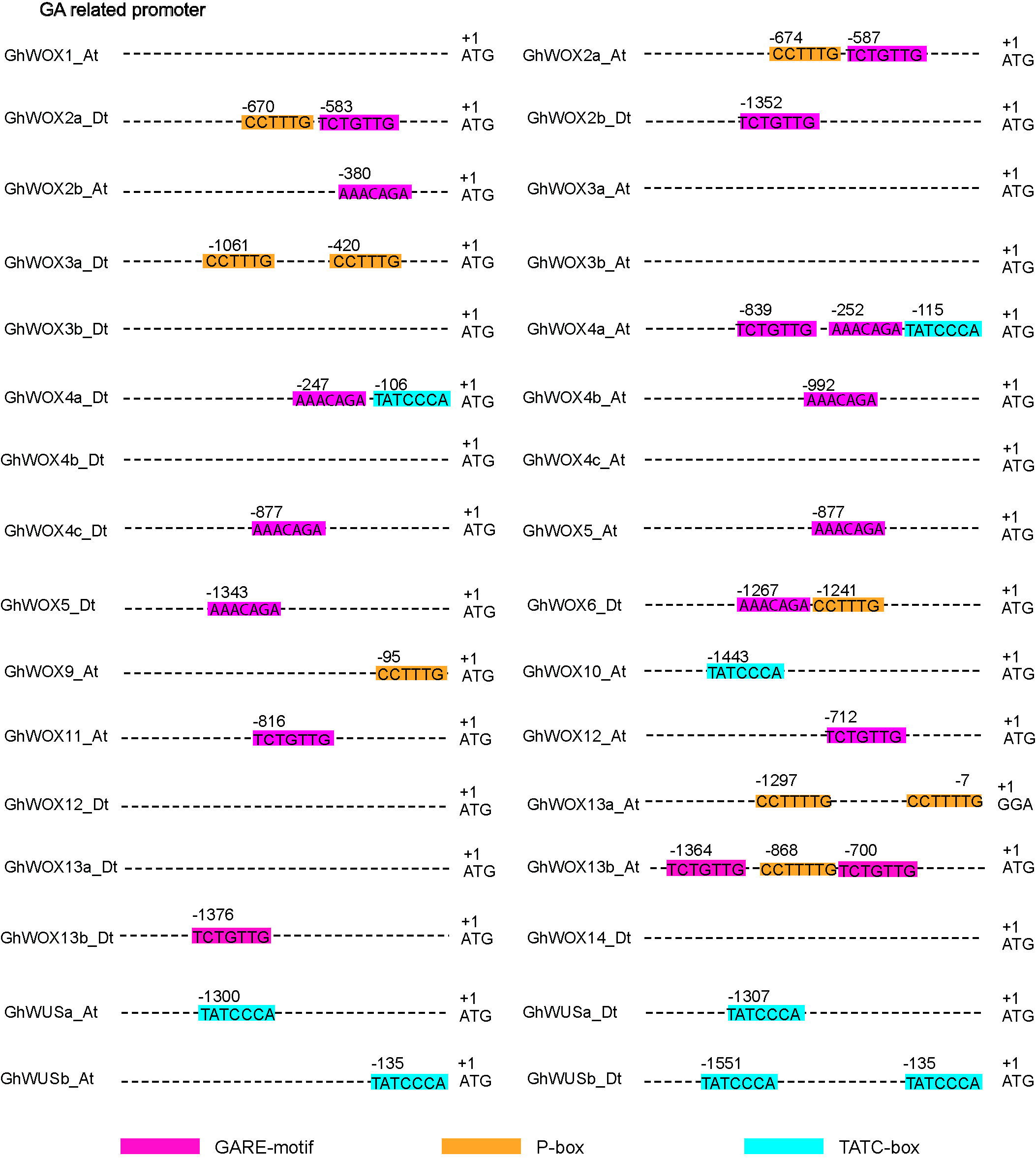
Supplementary Figure 1**. Identification of GA-related cis-elements in the promoter regions of the *GhWOX* genes.

**Supplementary Figure 2**. BR-related cis-elements identified in the *GhWOX* gene promoter regions.


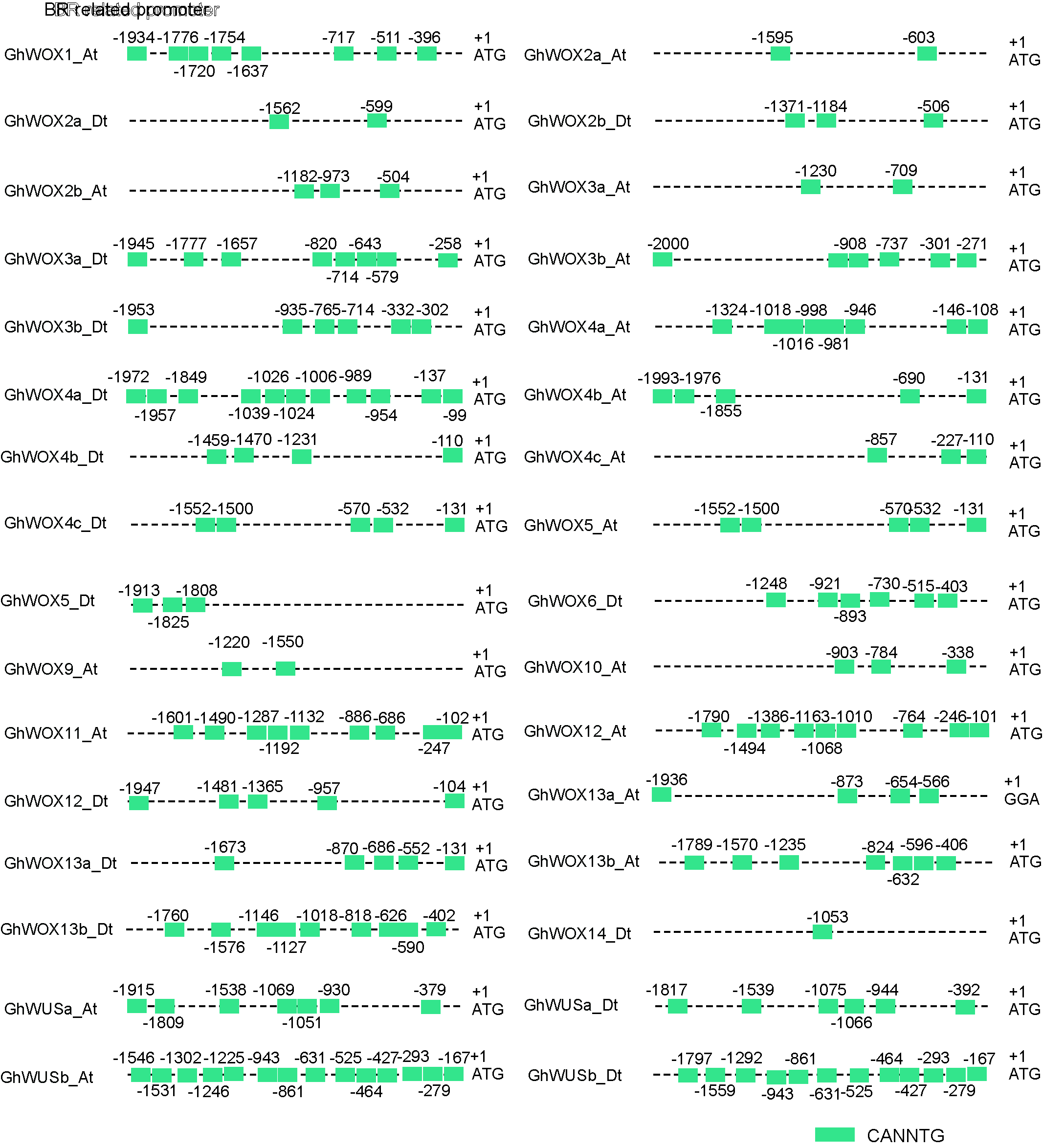


**Supplementary Figure 3**. Detection of auxin-related cis-elements in the *GhWOX* gene promoter regions.


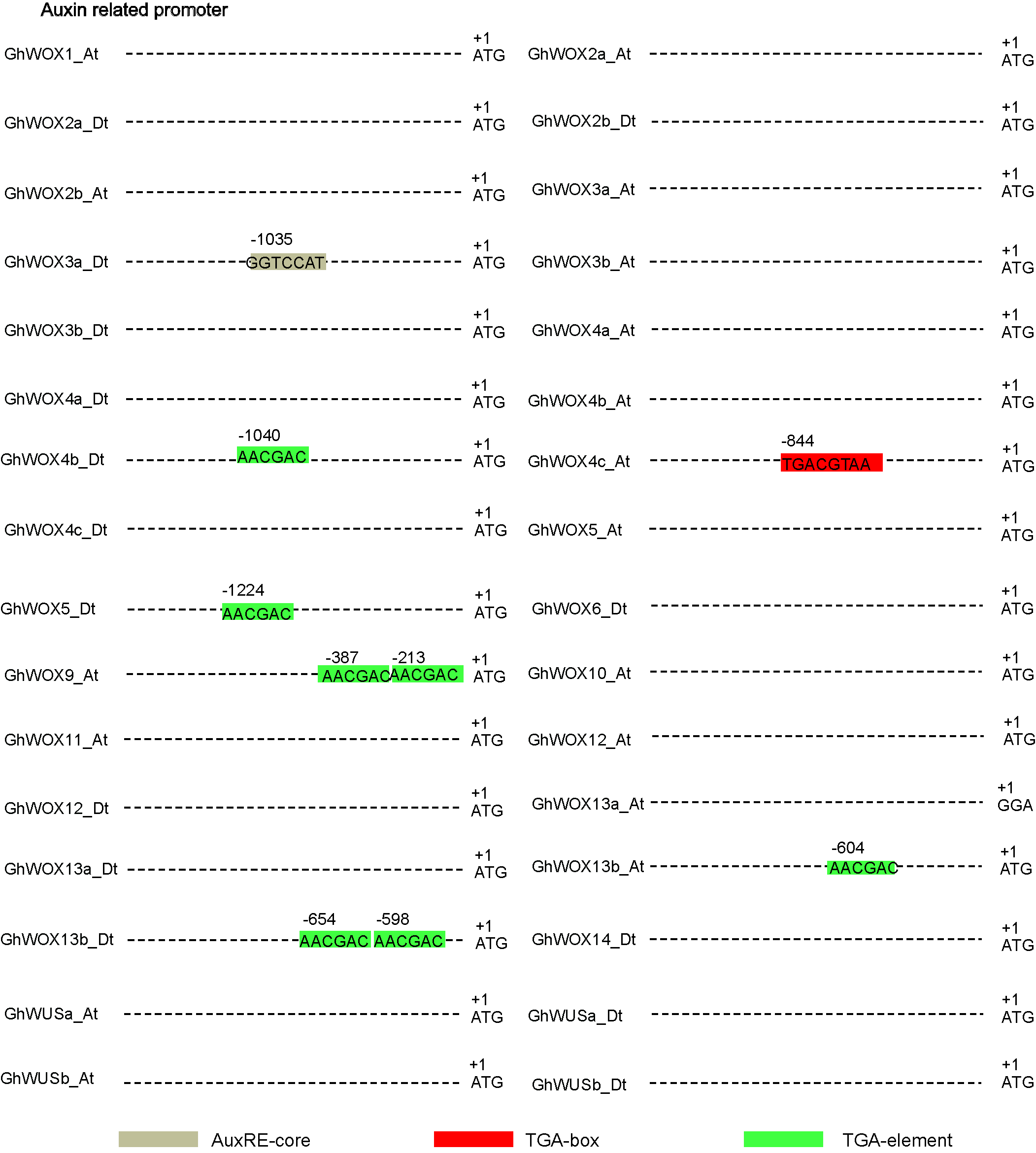

Supplement: Supplementary file 1 — Figure S1. Identification of putative GA-related cis-elements in the promoter regions of the GhWOX genes. Figure S2. Putative BR response elements identified in the GhWOX gene promoter regions. Figure S3. Detection of putative auxin-related cis-elements in the GhWOX gene promoter regions. (DOCX 3317 kb) [file 12870_2019_1892_MOESM1_ESM.docx]
