# Supplementary material for: Comprehensive analysis of WOX genes uncovers that WOX13 is involved in phytohormone-mediated fiber development in cotton
Source: BMC Plant Biol. 2019 Jul 15;19:312. doi: 10.1186/s12870-019-1892-x (PMC6632001; doi:10.1186/s12870-019-1892-x)
Supplement: Supplementary file 2 — Table S1. Analysis of the G. hirsutum WOX gene family members and their orthologs in the AA and DD genome cotton species. Table S2. Analysis of duplication events in the G. hirsutum WOX genes mapped to chromosomes. Table S3. PCR primers used for analysis of duplication events in the G. hirsutum WOX13 gene family members mapped to chromosomes. (DOC 107 kb) [file 12870_2019_1892_MOESM2_ESM.doc]

**Supplementary Table 1**. Analysis of the *G. hirsutum* *WOX* gene family members and their orthologs in the AA and DD genome cotton species

| **Gene ID** | **Length (aa)** | **Gene**  **name** | **Ortholog** | **Length (aa)** | **Gene name** |
| --- | --- | --- | --- | --- | --- |
| Gh_A12G2429.1 | 364 | GhWOX1_At | Ga12G0086 | 364 | GaWOX1a |
| Gh_A13G1983.1 | 248 | GhWOX2a_At | Ga07G1049 | 248 | GaWOX2a |
| Gh_A07G0882.1 | 244 | GhWOX2b_At | [Ga13G2770](https://cottonfgd.org/sequenceserver/#Query_1_hit_1) | 246 | GaWOX2b |
| Gh_A03G0301.1 | 200 | GhWOX3a_At | Ga01G2409 | 200 | GaWOX3a |
| Gh_A05G0852.1 | 232 | GhWOX3b_At | Ga05G1053 | 232 | GaWOX3b |
| Gh_A05G1768.1 | 218 | GhWOX4a_At | Ga05G2173 | 215 | GaWOX4a |
| Gh_A02G0851.1 | 200 | GhWOX4b_At | NA | NA | NA |
| Gh_A01G0998.1 | 217 | GhWOX4c_At | Ga01G1410 | 214 | GaWOX4c |
| Gh_A10G0270.1 | 343 | GhWOX5_At | Ga10G0004 | 187 | GaWOX5 |
| Gh_A05G1334.1 | 266 | GhWOX9_At | Ga10G2777 | 345 | GaWOX9a |
| NA | NA | NA | Ga05G1688 | 377 | GaWOX9b |
| Gh_A08G0247.1 | 215 | GhWOX10_At | Ga08G0340 | 215 | GaWOX10 |
| Gh_A13G1402.1 | 248 | GhWOX11_At | Ga13G2025 | 249 | GaWOX11 |
| Gh_A11G2676.1 | 252 | GhWOX12_At | Ga11G0392 | 254 | GaWOX12 |
| Gh_A07G1563.1 | 287 | GhWOX13a_At | Ga14G0211 | 287 | GaWOX13a |
| Gh_A02G1705.1 | 245 | GhWOX13b_At | Ga02G0019 | 244 | GaWOX13b |
| NA | NA | NA | Ga11G0090 | 223 | GaWOX14 |
| Gh_A10G0884.1 | 289 | GhWUS1a_At | Ga10G1993 | 246 | GaWUS1a |
| Gh_A12G0552.1 | 279 | GhWUS1b_At | Ga12G2478 | 316 | GaWUS1b |
| NA | NA | NA | Cotton_D_gene_10030619 | 364 | GrWOX1 |
| Gh_D07G0951.1 | 246 | GhWOX2a_Dt | Cotton_D_gene_10025280 | 246 | GrWOX2 |
| Gh_D13G2382.1 | 244 | GhWOX2b_Dt | NA | NA | NA |
| Gh_D03G1275.1 | 202 | GhWOX3a_Dt | Cotton_D_gene_10006622 | 201 | GrWOX3a |
| Gh_D05G3885.1 | 230 | GhWOX3b_Dt | Cotton_D_gene_10032237 | 230 | GrWOX3b |
| Gh_D05G1962.1 | 215 | GhWOX4a_Dt | Cotton_D_gene_10011476 | 215 | GrWOX4a |
| Gh_D01G1055.1 | 217 | GhWOX4b_Dt | Cotton_D_gene_10038872 | 217 | GrWOX4b |
| Gh_D05G1962.1 | 217 | GhWOX4c_Dt | Cotton_D_gene_10039447 | 200 | GrWOX4c |
| Gh_D02G0901.1 | 200 | GhWOX5_Dt | Cotton_D_gene_10009328 | 188 | GrWOX5 |
| Gh_D01G1463.1 | 291 | GhWOX6_Dt | Cotton_D_gene_10014360 | 286 | GrWOX6 |
| NA | NA | NA | Cotton_D_gene_10033710 | 377 | GrWOX9a |
| NA | NA | NA | Cotton_D_gene_10031402 | 343 | GrWOX9b |
| NA | NA | NA | Cotton_D_gene_10001321 | 249 | GrWOX11 |
| Gh_D13G1717.1 | 249 | GhWOX12_Dt | Cotton_D_gene_10027462 | 252 | GrWOX12 |
| Gh_D07G1730.1 | 247 | GhWOX13a_Dt | Cotton_D_gene_10030366 | 254 | GrWOX13a |
| Gh_D03G0014.1 | 246 | GhWOX13b_Dt | Cotton_D_gene_10011892 | 246 | GrWOX13b |
| Gh_D08G0336.1 | 214 | GhWOX14_Dt | Cotton_D_gene_10001458 | 215 | GrWOX14a |
| NA | NA | NA | Cotton_D_gene_10004194 | 198 | GrWOX14b |
| Gh_D10G0866.1 | 289 | GhWUS1a_Dt | Cotton_D_gene_10001209 | 289 | GrWUS1a |
| Gh_D12G0569.1 | 279 | GhWUS1b_Dt | Cotton_D_gene_10040315 | 279 | GrWUS1b |

**Supplementary Table 2**. Analysis of duplication events in the *G. hirsutum* *WOX* genes mapped to chromosomes.

| **Gene ID** | **Gene name** | **Duplication type** |
| --- | --- | --- |
| Gh_A13G1983.1 | GhWOX2a_At | WGD/ segmental |
| Gh_D07G0951.1 | GhWOX2a_Dt | WGD/ segmental |
| Gh_A03G0301.1 | GhWOX3a_At | WGD/ segmental |
| Gh_D03G1275.1 | GhWOX3a_Dt | WGD/ segmental |
| Gh_A05G0852.1 | GhWOX3b_At | WGD/ segmental |
| Gh_D05G3885.1 | GhWOX3b_Dt | WGD/ segmental |
| Gh_D05G1962.1 | GhWOX4a_Dt | WGD/ segmental |
| Gh_A05G1768.1 | GhWOX4a_At | WGD/ segmental |
| Gh_D01G1055.1 | GhWOX4b_Dt | WGD/ segmental |
| Gh_A02G0851.1 | GhWOX4b_At | WGD/ segmental |
| Gh_D02G0901.1 | GhWOX5_Dt | WGD/ segmental |
| Gh_A01G0998.1 | GhWOX4c_At | WGD/ segmental |
| Gh_D05G1962.1 | GhWOX4c_Dt | WGD/ segmental |
| Gh_A13G1402.1 | GhWOX11_At | WGD/ segmental |
| Gh_D13G1717.1 | GhWOX12_Dt | WGD/ segmental |
| Gh_A11G2676.1 | GhWOX12_At | dispersed |
| Gh_D07G1730.1 | GhWOX13a_Dt | WGD/ segmental |
| Gh_A07G1563.1 | GhWOX13a_At | WGD/ segmental |
| Gh_D10G0866.1 | GhWUS1a_Dt | WGD/ segmental |
| Gh_A10G0884.1 | GhWUS1a_At | WGD/ segmental |
| Gh_A12G0552.1 | GhWUS1b_At | WGD/ segmental |
| Gh_D12G0569.1 | GhWUS1b_Dt | tandem |

WGD, whole genome duplication

**Supplementary Table 3**. PCR primers used for analysis of duplication events in the *G. hirsutum* *WOX13* gene family members mapped to chromosomes.

| **Gene ID** | **Sense primer sequence** | **Antisense primer sequence** |
| --- | --- | --- |
| GhWOX13b_At | TCTTCTCTCTGGGCAGAACC | ATGTTGTAGCTCCCAGGCAT |
| GhWOX13b_Dt | GACTGGAACTCCAAGCAAGC | CGGTGACTCGACTTCTGTCT |
| GhWOX13a_At | AGAAGTTGAGTCGCCAAGGA | CCTGCCATGCTTGGTCATAA |
| GhWOX13a_Dt | CGGAAGCAGATTGCCGTTTA | ATTTCCCAGTCTCCCACCTG |
| UBQ7 | GGCATTCCACCTGACCAACAA | CCGCATTAGGGCACTCTTTTC |
